# Early Prediction for Prediabetes and Type 2 Diabetes Using the Genetic Risk Score and Oxidative Stress Score

**DOI:** 10.3390/antiox11061196

**Published:** 2022-06-17

**Authors:** Ximei Huang, Youngmin Han, Kyunghye Jang, Minjoo Kim

**Affiliations:** 1 Department of Food and Nutrition, College of Life Science and Nano Technology, Hannam University, Daejeon 34054, Korea; 20204263@hnu.kr; 2 Institute for Health Promotion, Graduate School of Public Health, Yonsei University, Seoul 03722, Korea; ymhan@yuhs.ac; 3 Nakdonggang National Institute of Biological Resources, Sangju, Gyeongsangbuk-do 37242, Korea; jkh2761@nnibr.re.kr

**Keywords:** genetic risk score, oxidative stress score, prediabetes, type 2 diabetes

## Abstract

We aimed to use a genetic risk score (GRS) constructed with prediabetes and type 2 diabetes-related single nucleotide polymorphisms (SNPs) and an oxidative stress score (OSS) to construct an early-prediction model for prediabetes and type 2 diabetes (T2DM) incidence in a Korean population. The study population included 549 prediabetes and T2DM patients and 1036 normal subjects. The GRS was constructed using six prediabetes and T2DM-related SNPs, and the OSS was composed of three recognized oxidative stress biomarkers. Among the nine SNPs, six showed significant associations with the incidence of prediabetes and T2DM. The GRS was profoundly associated with increased prediabetes and T2DM (OR = 1.946) compared with individual SNPs after adjusting for age, sex, and BMI. Each of the three oxidative stress biomarkers was markedly higher in the prediabetes and T2DM group than in the normal group, and the OSS was significantly associated with increased prediabetes and T2DM (OR = 2.270). When BMI was introduced to the model with the OSS and GRS, the area under the ROC curve improved (from 69.3% to 70.5%). We found that the prediction model composed of the OSS, GRS, and BMI showed a significant prediction ability for the incidence of prediabetes and T2DM.

## 1. Introduction

The prevalence of type 2 diabetes mellitus (T2DM) has been increasing and evolving rapidly worldwide in recent years, highlighting the importance of preventing T2DM in high-risk individuals. Prediabetes is the early phase of diabetes, encompassing impaired fasting glucose (IFG) and impaired glucose tolerance (IGT) [1]. Prediabetes is considered the critical stage because studies have suggested that the treatment of prediabetes could prevent or reduce the further progression of diabetes and diabetes-related complications [2].

Oxidative stress has been widely linked with the occurrence and development of diabetes and its associated complications [3]. Several studies have shown that increased oxidative stress is a causative factor in developing insulin resistance (IR), β-cell dysfunction, IFG, and IGT [4,5]. In addition, the levels of oxidative stress markers are elevated in patients with T2DM [6]. Malondialdehyde (MDA) and 8-epi-prostaglandin F_2α_ (8-epi-PGF_2α_) are accepted as reliable biomarkers of oxidative stress; indeed, previous studies have shown that 8-epi-PGF_2α_ is significantly associated with IR and prediabetes [7]. Furthermore, circulating oxidized (ox)-low-density lipoprotein (LDL) can be used as a crucial biomarker of oxidative stress and is a strong indicator of the risk of IR [8]. According to previous studies, the oxidative stress score (OSS), calculated as a weighted sum of MDA, ox-LDL, and 8-epi-PGF_2α_, is more accurate for reflecting the overall oxidative stress state than individual biomarkers [9].

In recent years, genome-wide association studies (GWASs) have discovered several T2DM-associated susceptibility single nucleotide polymorphism (SNP) loci in Western populations [10]. Given the remarkable genetic variations between the Korean and Western populations, we identified T2DM-related SNP loci in a Korean population with the Korean Chip (K-CHIP), which is a suitable tool for analyzing Korean population-specific SNPs associated with disease occurrence [11]. Furthermore, the genetic risk score (GRS), a reliable risk predictor for T2DM, can be computed by summarizing variation across multiple genetic variants [12,13]. Thus, we constructed a GRS to generate an overall score for predicting prediabetes and T2DM in a Korean population. In addition, the diagnosis of prediabetes or T2DM was based on fasting serum glucose and HbA1c levels to predict prediabetes and T2DM accurately. Therefore, this study aimed to assess the prediction model for prediabetes and T2DM composed of the OSS, GRS, and T2DM-related risk factors. Additionally, we further determined the optimal cutoff thresholds of risk factors for the incidence of prediabetes and T2DM.

## 2. Materials and Methods

### 2.1. Study Population

In total, 1585 normal, prediabetes, and T2DM study participants between the ages of 20 and 80 years were recruited from the National Leading Research Laboratory of Clinical Nutrigenetics/Nutrigenomics at Yonsei University and the National Health Insurance Corporation of Ilsan Hospital in Goyang, Republic of Korea (2014–2019). The diagnosis of prediabetes and T2DM was based on fasting serum glucose (≥126 mg/dL or 100–125 mg/dL, respectively) and HbA1c levels (≥6.5% or 5.7–6.4%, respectively). The exclusion criteria included a current diagnosis and/or history of cardiovascular disease, liver disease, renal disease, pancreatitis, or cancer, and medication use. All the study participants provided written informed consent, and the Institutional Review Board of Hannam University (20-04-06-0114) approved the study protocol, which complied with the Declaration of Helsinki.

### 2.2. SNP Selection and SNP Chip Analysis

According to a GWAS and a previous study, nine SNPs were established at IFG and T2DM loci showing the strongest associations with glycemic traits [9]. SNP chip analysis was performed using an Axiom^®^ Korean Biobank Array 1.1. The K-CHIP, available from the K-CHIP consortium, was used to generate the genotype data. The K-CHIP was created by the Center for Genome Science at the Korea National Institute of Health (4845-301, 3000-3031). The specific procedures were documented in a previous study [14].

### 2.3. GRS and OSS Construction

We constructed a GRS utilizing six SNPs from the nine SNPs that showed statistical significance (*p* < 0.05) and a consistent effect direction in the Korean population. Based on Bonferroni’s threshold (*p* < 0.006, 0.05 divided by 9), five SNPs remained after multiple testing. All the SNPs with a nominal *p* value < 0.05 were included in the following risk score analysis to maximize statistical power. A logistic regression evaluating the association between the number of risk alleles and prediabetes and T2DM status was used to examine the estimate. The GRS was determined by multiplying each estimated β-coefficient by the number of risk alleles (0, 1, or 2). The combined data from three oxidative stress biomarkers, MDA, ox-LDL, and 8-epi-PGF_2α_, were used to calculate the OSS. Continuous variables were categorized into low, medium, and high according to the tertile values of each concentration, and points (0, 1, or 2) were assigned based on tertile categorization. A higher score indicates a greater oxidative stress state. In this research, the OSS is the sum of the tertile (T1 = 0, T2 = 1, and T3 = 2) concentrations of ox-LDL (T1, lowest through 36.34 U/L; T2, 36.34 through 51.76 U/L; and T3, 51.76 U/L through highest), MDA (T1, lowest through 7.44 nmol/mL; T2, 7.44 through 9.41 nmol/mL; and T3, 9.41 nmol/mL through highest), and 8-epi-PGF_2α_ (T1, lowest through 1117.49 pg/mg creatinine; T2, 1117.49 through 1615.60 pg/mg creatinine; and T3, 1615.60 pg/mg creatinine through highest). The OSS was calculated in the same way as the GRS. The weights for the OSS β-coefficient were calculated using the coefficient estimates for each of the components obtained from the regression model.

### 2.4. Laboratory Assessments

Detailed anthropometry and laboratory assessment information has been previously reported [15]. The participants’ body mass index (BMI) and waist-to-hip ratio were calculated via their weight and height, and waist circumference. After at least 20 min of rest, blood pressure (BP) was measured using an automated BP monitor. The venous blood samples and urine samples following an overnight fast of at least 12 h were collected and stored at −80 and −20 °C, respectively. To check the lipid profile, commercial kits and an autoanalyzer were used to measure serum triglyceride (TG), total cholesterol, and high-density lipoprotein (HDL) cholesterol levels, and the Friedewald formula was applied to calculate LDL cholesterol levels. The fasting serum glucose level and glucose-related indicators, including insulin, homeostatic model assessment for insulin resistance (HOMA-IR) score, and HbA1c, were also measured. Commercial assay kits were used to detect the urinary 8-epi-PGF_2α_ and plasma MDA concentrations [16].

### 2.5. Statistical Analysis

IBM SPSS statistics v.26.0 was used for all statistical analyses. Skewed variables were log-transformed, and a two-tailed *p* value < 0.05 was considered statistically significant. An independent *t* test was performed to compare parameters between the two groups. The logistic regression model derived the odds ratio (OR) and 95% confidence interval (CI). The frequency of the risk allele was determined using a chi-square test. A general linear model UNIANOVA was utilized to adjust for confounding variables, and a stepwise regression analysis was conducted to determine representative parameters of prediabetes and T2DM incidence. The optimal cutoff threshold for each risk factor was determined using a receiver operating characteristic (ROC) curve analysis. 

## 3. Results

### 3.1. Clinical Characteristics

The clinical and biochemical characteristics of the prediabetes and T2DM group (*n* = 549) and the control group (*n* = 1036) are shown in Table 1. After adjusting for age, sex, and BMI, the prediabetes and T2DM group had a significantly higher waist circumference, systolic and diastolic BP, fasting glucose, insulin, HOMA-IR, HbA1c, TG, high-sensitivity C-reactive protein (hs-CRP), adiponectin, gamma-glutamyl transferase (γGTP), MDA, ox-LDL, and 8-epi-PGF_2α_ than those of the control group (Table 1).

### 3.2. Association of the SNP Loci with Prediabetes and T2DM and Construction of the GRS

Using nine SNPs from six loci, a GRS was constructed using six SNPs that had statistical significance and a consistent effect direction in a Korean population (Table 2). Six SNPs (*GCKR* rs1260326; *SLC30A8* rs11558471; *CDKN2A/B* rs10811661; and *MTNR1B* rs1387153, rs2166706, and rs10830963) were prominently associated with prediabetes and T2DM after adjusting for age, sex, and BMI, and rs10811661 in *CDKN2A/B* exhibited the most powerful association with increased prediabetes and T2DM (OR = 1.346, 95% CI = 1.143–1.585). Furthermore, the GRS was markedly associated with increased prediabetes and T2DM (OR = 1.946, 95% CI = 1.545–2.453) compared with individual SNPs after adjusting for age, sex, and BMI (Table 2).

### 3.3. Associations between the OSS and Incidence of Prediabetes and T2DM

The levels of oxidative stress biomarkers, including MDA, ox-LDL, and 8-epi-PGF_2α_, were significantly higher in the prediabetes and T2DM group (Table 1). The OSS was the sum of the tertile concentrations of these three oxidative stress biomarkers. Logistic regression analysis was used to examine the associations of oxidative stress biomarkers with prediabetes and T2DM; significant associations were found between prediabetes and T2DM incidence and MDA (OR = 1.711. 95% CI = 1.478–1.980), ox-LDL (OR = 1.252, 95% CI = 1.088–1.440), and 8-epi-PGF_2α_ (OR = 1.135, 95% CI = 0.992–1.300) (Table 3). Additionally, the OSS showed a strongly significant association with the incidence of prediabetes and T2DM, with an OR of 2.270 (*p* = 3.0244 × 10^−16^).

### 3.4. Optimal cutoff Thresholds of Risk Factors for the Incidence of Prediabetes and T2DM

**Figure 1:**
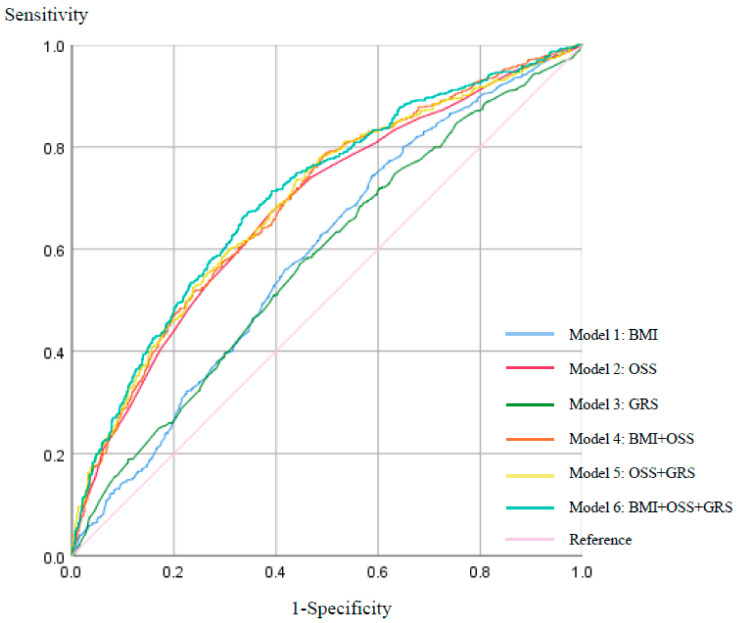
ROC curves for BMI, OSS, and GRS.

Among the diabetes-related parameters, we found BMI to be the most relevant factor for increasing the risk of prediabetes and T2DM except fasting glucose, insulin, HOMA-IR score, and HbA1c through stepwise linear regression analysis. The optimal cutoff threshold of risk factors for the incidence of prediabetes and T2DM, including BMI, OSS, and GRS, was identified through the regression test. The area under the ROC curve (AUROC) (Table S1) was 0.561 ± 0.619 (*p* < 0.001) for BMI (Model 1), 0.652 ± 0.709 (*p* < 0.001) for OSS (Model 2), 0.549 ± 0.608 (*p* < 0.001) for GRS (Model 3), 0.663 ± 0.718 (*p* <0.001) for BMI and OSS (Model 4), and 0.666 ± 0.721 (*p* < 0.001) for OSS and GRS (Model 5). When the BMI was introduced to the model along with the OSS and GRS, the AUROC improved; the AUROC increased from 69.3% to 70.5% (Table S1). A BMI of 24.197 (sensitivity = 0.570, specificity = 0.568), an OSS of 1.322 (sensitivity = 0.615, specificity = 0.657), and a GRS of 1.556 (sensitivity = 0.561, specificity = 0.558) were the optimal cutoff thresholds (Figure 1); that is, a Korean population having a BMI above 24.197 kg/m^2^ and an OSS greater than 1.322 with a GRS greater than 1.556 were more likely to have prediabetes and T2DM. According to regression analyses, GRS, OSS, and BMI were all linked to the incidence of prediabetes and T2DM (Table S1).

## 4. Discussion

In the current study, we constructed a GRS using six susceptibility SNPs for prediabetes and T2DM in a Korean population and confirmed the influence of the GRS on the incidence of prediabetes and T2DM. The OSS, composed of MDA, ox-LDL, and 8-epi-PGF_2α_, showed a stronger ability to predict the incidence of prediabetes and T2DM than individual components. A high OSS means that oxidative stress is predominant and that the risk of prediabetes and T2DM is significantly increased. Additionally, the prediction model with BMI, OSS, and GRS greatly improved the prediabetes and T2DM incidence prediction model.

Several studies have shown that increased oxidative stress plays a crucial role in the pathogenesis and progression of T2DM [17,18]. Oxidative stress occurs due to the imbalance between the presence of reactive oxygen species (ROS) and antioxidants, which are influenced by both modifiable extrinsic factors and unmodifiable intrinsic factors [19]. Since oxidative stress is a complicated process with multiple factors, other studies of chronic diseases have focused on establishing effective oxidative stress scoring systems to measure oxidative stress status. Therefore, several previous studies have constructed an oxidative balance score (OBS), a comprehensive measure of combined pro- and antioxidant exposure status [20,21]. Previous research has shown that having a greater OBS is related to a lower risk of prostate cancer and colorectal neoplasia [22,23], and the OBS can be a comprehensive measure of oxidative stress [24]. Although the OBS has been widely used in epidemiological studies, previous studies have also indicated that a limitation of the OBS is that it fails to consider endogenous factors associated with oxidative stress [20,22,25]. Kaiming Zhang et al. [26] established a systematic oxidative stress score (SOS) based on biochemical indicators of systematic oxidative stress, including serum creatinine (CRE), serum albumin (ALB), total bilirubin (TBIL), lactate dehydrogenase (LDH), and blood urea nitrogen (BUN). They found that SOS can be an independent prognostic indicator of operable breast cancer. Yinghao Cao et al. [27] designed a colorectal cancer-integrated oxidative stress score (CIOSS) based on a combination of available oxidative stress indexes, including ALB, direct bilirubin (DBIL), and BUN, and found that the CIOSS had a powerful predictive performance in colorectal cancer patients. Accordingly, approaches that use a combination of factors to create comprehensive scores for oxidative stress showed a more statistically significant association with disease risk than approaches that use a single factor. In the present study, the prediabetes and T2DM group exhibited significantly higher concentrations of MDA, ox-LDL, and 8-epi-PGF_2α_ than those of the control group. Moreover, the OSS comprised MDA, ox-LDL, and 8-epi-PGF_2α_, which are oxidative stress biomarkers that are significantly associated with T2DM. Therefore, in establishing a predictive model for prediabetes and T2DM, the OSS model, whose AUC was 0.680, showed better predictive performance than the BMI model in prediabetes and T2DM. In addition, Park et al. [28] have previously proposed an OSS that solely used three intrinsic biomarkers of oxidative stress, namely MDA, ox-LDL, and 8-epi-PGF_2α_, and showed that a higher OSS was associated with a higher risk of obesity. Therefore, in this study, the OSS using biomarkers can accurately reflect the internal unmodifiable factors of oxidative stress and can act as an indicator to predict the incidence of prediabetes and T2DM.

It is universally acknowledged that obesity is the leading risk factor for many chronic diseases, such as cardiovascular disease (CVD), T2DM, hypertension (HTN), and coronary heart disease. T2DM has the strongest association with obesity among these diseases. Obesity significantly increases the incidence of T2DM in individuals: among individuals over age 65, both men and women in the BMI range of 25 to 29.99 kg/m^2^ had an increased risk of developing T2DM, with 30% and 10% greater risks, respectively [29]. The Nurses’ Health Study highlighted the importance of weight management since T2DM risk increases with BMI above 25 kg/m^2^ [30]. The current study led to a similar conclusion, where a BMI ≥ 24.197 kg/m^2^ was associated with the risk of prediabetes and T2DM in the Korean population. In the Framingham Offspring Study, a simple clinical model using BMI efficiently identified subjects at elevated risk of T2DM [31]; this result suggested that BMI has a strong predictive ability for T2DM. In addition to BMI, the levels of lipids, especially TG, and obesity are strongly associated with T2DM prevalence and incidence [32]. Higher serum TG concentrations were associated with higher plasma glucose, HbA1c, insulin, and HOMA-IR [33]. A retrospective cohort study revealed that the TG level could be an independent risk factor and prediction factor of T2DM [34]. Hs-CRP is a well-studied and well-known biomarker of inflammation that plays a role in the development of T2DM [35]. For example, a prospective study of a general Japanese population showed that an elevated hs-CRP level is an independent predictor of diabetes after adjustment for comprehensive risk factors [36]. Adiponectin, a hormone secreted by adipocytes with putative antiatherogenic and anti-inflammatory properties, plays a significant regulatory role in insulin action and sensitivity [37,38]. Indeed, it has been reported that the plasma adiponectin level is inversely associated with BMI, and low plasma adiponectin concentrations are directly associated with obesity and T2DM in different ethnic groups [39,40]. γGTP, a liver enzyme, is not only a well-known marker for alcohol abuse or liver disturbances but is also a marker for the development of CVD, metabolic syndrome (MetS), and T2DM independent of alcohol consumption [41,42,43]. A limited number of prospective studies have found that other liver enzymes, including glutamic oxaloacetic transaminase (GOT) and glutamic pyruvic transaminase (GPT), were significantly associated with T2DM incidence [44,45]. In contrast, in a study of the Japanese population, neither GOT nor GPT was associated with diabetes risk [44]. Moreover, a large community-based cohort study confirmed that elevated levels of γGTP were a better indicator of diabetes than either GOT or GPT, indicating that γGTP may have the strongest association with the risk of diabetes [46]. This result is consistent with the present study. In the current study, γGTP levels were significantly higher in the prediabetes and T2DM group than in the control group after adjusting for conventional risk factors, while GOT and GPT were nonsignificant.

Developing prediction models to identify individuals at risk of early onset of T2DM is critical for developing measures to prevent the onset of T2DM. Polygenic risk scores have a certain attraction as a risk predictor because the genetic code remains unchanged throughout the course of life [10]. The present study demonstrated that a GRS based on six diabetes-related SNPs greatly improved the risk prediction of prediabetes and T2DM compared to a single SNP after adjusting for clinical risk factors. This result is in line with the findings of other studies, wherein the GRS evaluated the cumulative effects of genetic factors [47,48]. While a single GRS model (Model 3) showed a lower predictive ability for prediabetes and T2DM compared to the single models of OSS (Model 2) and BMI (Model 1), combining the three factors (Model 6) prompted a modest increase in the current study. Miranda-Lora et al. [47] suggested that the GRS alone or in combination with T2DM clinical factors increased the power of risk prediction models. Conclusively, the GRS will be helpful for future practical use in improving the prediction of prediabetes and T2DM, and additional susceptibility SNPs will contribute to further strengthening risk prediction models. The *CDKN2A/B* locus, the cyclin-dependent kinase inhibitor 2 A/B gene at chromosome 9p21, influences diabetes risk through islet gene expression, β-cell proliferation, and non-islet mechanisms [49]. A large replication study of 6719 Asians suggested that many of the genetic variants associated with T2DM in Europeans have important but differential associations in Asians and confirmed the associations of *SLC30A8* and *CDKN2A/2B* with an increased risk of T2DM [50]. Furthermore, Ying Wu et al. [51] revealed a strong association between *CDKN2A/B* rs10811661 and T2DM and IFG in Chinese individuals, with a marginally higher OR than that observed in Europeans. This significant association between rs10811661 and prediabetes and T2DM was also found in the present study. GWAS and many gene studies have shown that the genetic variation in glucokinase regulatory protein (*GCKR*) is associated with serum TGs, insulin secretion, and glycogen metabolism [52]. Common functional variants of *GCKR*, namely rs780094 and rs1260326, were shown to be associated with T2DM and MetS and were perceived as potential T2DM susceptibility variants [53,54]. In this research, only rs1260326 in *GCKR* was detected in the Korean population and showed a strong association with the risk of prediabetes and T2DM. The *SLC30A8* gene encodes a pancreas-restricted zinc transporter (ZnT8), which is associated with a high risk of T2DM [55]. In a recent study, rs11558471 in *SLC30A8* was shown to be a functional variant associated with IR and fasting glucose [56]. The melatonin receptor 1B gene (*MTNR1B*), encoding the melatonin receptor MT2, is a susceptibility gene associated with glucose concentrations and T2DM [57]. The results of this study reconfirmed that the abovementioned six SNPs were strongly associated with prediabetes and T2DM risk in the Korean population.

Nevertheless, there are also some discrepancies between this study and previous research. A previous study reported that *DGKB* (rs2191349) and *GCK* (rs1799884 and rs4607517) were significantly associated with IFG and T2DM in the Korean population [9], but no strong association was identified for those polymorphisms in the present study. Sara Moradipoor et al. [58] evaluated the gene expression levels in prediabetes and T2DM and observed that 17 genes displayed substantially higher expression in the T2DM group than in the prediabetes group, indicating differences in gene expression between prediabetes and T2DM conditions. In this study, the case group was made up of individuals with prediabetes or T2DM. Given the genetic difference between prediabetes and T2DM, significant T2DM-related SNP loci may not have the same susceptibility to prediabetes. Moreover, IFG or T2DM was diagnosed based only on fasting serum glucose levels in a previous study [9]. For a more accurate diagnosis, the present study used both fasting serum glucose and HbA1c levels together to diagnose prediabetes and T2DM. This strategy could explain why the correlation between the DGKB (rs2191349) and GCK (rs1799884 and rs4607517) polymorphisms and the prediabetes and T2DM group was not found in the current study. More studies on specific prediabetes and T2DM susceptibility genes in the Korean population are needed in the future.

Some limitations should be noted. First, we included only Korean populations; hence, our risk prediction model could have some limitations when applied to other ethnic groups. Second, a prospective cohort is needed to verify its predictive ability for general use. Moreover, the GRS included fewer SNPs likely to generate false positives; further studies are warranted to discover more susceptibility variants in Korean populations. However, the present study has several strengths. First, the diagnosis of prediabetes and T2DM was based on fasting serum glucose and HbA1c levels, which can enhance the accuracy of diagnosis. The HbA1c levels show the average blood sugar level over the past 2 to 3 months. When glucose builds up in the blood, it binds to the hemoglobin in red blood cells. The HbA1c test measures how much glucose is bound. Red blood cells live for about three months, so the test shows the average glucose level in the blood for the past three months. If the glucose levels have been high over recent weeks, the HbA1c level will be higher. Collectively, the current study includes the prediabetes or T2DM patients who are not only newly diagnosed but also have regularly high estimated average glucose levels. Based on these inclusion criteria, we narrowed down SNP loci significantly associated with the prediabetes and T2DM phenotypes in a Korean population from nine to six SNPs compared to the previous report [9]. Thus, our prediction model using the present GRS consisting of six SNPs is more accurate in predicting prediabetes or T2DM in a Korean population than the former one. Additionally, the current model involves the summation of internal unmodifiable markers of oxidative stress; hence, we created a total score for oxidative stress that uses multiple factors. Therefore, applying a scoring approach to Korean-specific genetic risk and oxidative stress is worthwhile used for predictive models of prediabetes or T2DM. In other words, this result suggests that the GRS and OSS provide a new approach for establishing a prediction model for prediabetes and T2DM.

## 5. Conclusions

The current findings suggest that individuals with a high OSS and a high GRS for prediabetes and T2DM are more likely to have an increased incidence of prediabetes and T2DM. Furthermore, the prediction model, which consisted of the GRS, OSS, and BMI, effectively predicted the incidence of prediabetes and T2DM. Therefore, these findings and weighting approaches are applicable to predicting prediabetes and T2DM in clinical practice and help provide novel pathways for the prediabetes and type 2 diabetes prediction model.

## Figures and Tables

**Table 1:** Clinical and biochemical characteristics of the normal versus prediabetes and T2DM groups.

	Normal (*n* = 1036)		Prediabetes and T2DM (*n* = 549)		*p*	*p’*
Male/Female (n, %)	367 (35.4)/669 (64.6)		272 (49.5)/277 (50.5)		<0.001	
Age (years)	47.0	±0.33	52.9	±0.39	<0.001	-
Waist (cm)	83.0	±0.25	86.4	±0.33	<0.001	0.004
Weight (kg)	63.3	±0.33	66.0	±0.43	<0.001	0.463
BMI (kg/m^2^)	23.8	±0.09	24.7	±0.13	<0.001	-
Systolic BP (mmHg)	119.5	±0.48	125.4	±0.66	<0.001	0.008
Diastolic BP (mmHg)	75.1	±0.35	78.5	±0.44	<0.001	0.048
Glucose (mg/dL) *^∮^*	87.1	±0.26	112.2	±1.00	<0.001	<0.001
Insulin (μIU/dL) *^∮^*	8.15	±0.13	9.24	±0.26	<0.001	<0.001
HOMA-IR *^∮^*	1.76	±0.03	2.59	±0.09	<0.001	<0.001
HbA1c (%) *^∮^*	5.43	±0.02	6.24	±0.04	<0.001	<0.001
Triglycerides (mg/dL) *^∮^*	113.1	±2.12	145.9	±3.56	<0.001	<0.001
Total cholesterol (mg/dL) *^∮^*	197.0	±1.08	202.6	±1.57	0.004	0.460
HDL cholesterol (mg/dL) *^∮^*	55.0	±0.42	52.0	±0.53	<0.001	0.147
LDL cholesterol (mg/dL) *^∮^*	119.7	±0.99	123.7	±1.45	0.032	0.883
hs-CRP (mg/L) *^∮^*	1.17	±0.10	1.56	±0.12	<0.001	0.001
Adiponectin (ng/mL) *^∮^*	6.65	±0.11	5.82	±0.15	<0.001	<0.001
γGTP (U/L) *^∮^*	22.9	±0.74	36.2	±3.07	<0.001	<0.001
GOT (U/L) *^∮^*	22.8	±0.23	24.7	±0.35	<0.001	0.266
GPT (U/L) *^∮^*	19.4	±0.31	22.5	±0.55	<0.001	0.159
Malondialdehyde (nmol/mL) *^∮^*	8.28	±0.07	10.4	±0.23	<0.001	<0.001
Ox-LDL (U/L) *^∮^*	45.2	±0.63	51.0	±0.87	<0.001	<0.001
8-epi-PGF_2α_ (pg/mg creatinine) *^∮^*	1559.7	±21.3	1597.3	±35.1	0.009	0.047

Mean ± SE. *^∮^* Tested by logarithmic transformation, *p* values derived were from an independent *t* test. *p’* values were derived from a general linear model UNIANOVA after adjusting for age, sex, and BMI.

**Table 2:** Association of nine SNP loci with the normal versus prediabetes and T2DM groups.

SNP	Nearby Gene ^a^	Risk Allele ^b^	RAF (Case/Control)	Unadjusted		Adjusted ^c^	
				*p* Value	OR (95% Cl)	*p* Value	OR (95% Cl)
rs1260326	*GCKR*	C	0.471/0.420	**0.006**	1.236 (1.063–1.436)	**0.002**	1.291 (1.100–1.515)
rs2191349	*DGKB*	T	0.696/0.674	0.208	1.106 (0.946–1.293)	0.158	1.127 (0.955–1.330)
rs1799884	*GCK*	T	0.201/0.176	0.075	1.189 (0.983–1.438)	0.163	1.154 (0.943–1.413)
rs4607517	*GCK*	A	0.239/0.219	0.206	1.121 (0.939–1.339)	0.332	1.097 (0.909–1.324)
rs11558471	*SLC30A8*	A	0.627/0.580	**0.011**	1.215 (1.046–1.412)	**0.019**	1.212 (1.033–1.422)
rs10811661	*GDKN2A/B*	T	0.595/0.537	**0.001**	1.282 (1.100–1.494)	**0.00037**	1.346 (1.143–1.585)
rs1387153	*MTNR1B*	T	0.463/0.417	**0.012**	1.211 (1.043–1.407)	**0.001**	1.301 (1.109–1.526)
rs2166706	*MTNR1B*	C	0.472/0.422	**0.007**	1.230 (1.059–1.428)	**0.001**	1.321 (1.126–1.548)
rs10830963	*MTNR1B*	G	0.485/0.440	**0.015**	1.198 (1.036–1.387)	**0.001**	1.307 (1.118–1.529)
GRS				**9.5294 × 10^−7^**	1.722 (1.386–2.141)	**1.644 × 10^−8^**	1.946 (1.545–2.453)

*p* values were derived from a logistic regression analysis. OR, odds ratio; 95% CI, 95% confidence interval; GRS, weighted genetic risk score. ^a^ Information in the original report is shown. ^b^ Risk allele reported in previous reports. ^c^ Adjusted for age, sex, and BMI. The GRS, including the SNPs with nominal significance (*p* < 0.05) shown in bold, was calculated.

**Table 3:** Associations of oxidative stress biomarkers with prediabetes and T2DM in a Korean population.

Oxidative Stress Biomarkers (Tertile)	*p* Value ^a^	OR (95% Cl) ^a^	*p* Value ^b^	OR (95% Cl) ^b^
MDA (nmol/mL) ^c^	2.3297 × 10^−13^	1.720 (1.488–1.989)	5.8456 × 10^−13^	1.711 (1.478–1.980)
Ox-LDL (U/L) ^c^	0.000055	1.327 (1.156–1.522)	0.002	1.252 (1.088–1.440)
8-epi-PGF_2α_ (pg/mg creatinine) ^c^	0.042	1.149 (1.005–1.314)	0.066	1.135 (0.992–1.300)
OSS	3.3791 × 10^−18^	2.372 (1.952–2.881)	3.0244 × 10^−16^	2.270 (1.865–2.764)

*p* values derived from a logistic regression analysis. OR, odds ratio; 95% CI, 95% confidence interval; OSS, oxidative stress score. ^a^ Adjusted for age and sex. ^b^ Adjusted for age, sex, and BMI. ^c^ Tested by logarithmic transformation.

## Data Availability

The data that support the findings of this study are available on request from the corresponding author.

## Supplementary Materials

The following supporting information can be downloaded at: https://www.mdpi.com/article/10.3390/antiox11061196/s1. Table S1. Odds ratios for the risk factors for the incidence of prediabetes.
