# Supplementary material for: Early Prediction for Prediabetes and Type 2 Diabetes Using the Genetic Risk Score and Oxidative Stress Score
Source: Antioxidants (Basel). 2022 Jun 17;11(6):1196. doi: 10.3390/antiox11061196 (PMC9231325; doi:10.3390/antiox11061196)
Supplement: Supplementary file 1 [file antioxidants-11-01196-s001.zip › antioxidants-1721335-supplementary.pdf]

Table S1. Odds ratios for risk factors for the incidence of prediabetes and T2DM in a Korean population.

	OR (95% CI)	*p* Value
Model 1		
BMI	1.112 (1.074–1.151)	2.2823×10^{-9}
AUROC	0.590 (0.561–0.619)	5.8534×10^{-9}
Model 2		
OSS	2.875 (2.389–3.460)	4.9763×10^{-29}
AUROC	0.680 (0.652–0.709)	1.5722×10^{-31}
Model 3		
GRS	1.722 (1.386–2.141)	9.5294×10^{-7}
AUROC	0.578 (0.549–0.608)	3.7834×10^{-7}
Model 4		
BMI	1.087 (1.048–1.127)	0.000009
OSS	2.740 (2.272–3.304)	4.793×10^{-26}
AUROC	0.691 (0.663–0.718)	4.3889×10^{-35}
Model 5		
OSS	2.942 (2.435–3.554)	5.1412×10^{-29}
GRS	1.724 (1.376–2.161)	0.000002
AUROC	0.693 (0.666–0.721)	5.729×10^{-36}
Model 6		
BMI	1.088 (1.048–1.130)	0.000010
OSS	2.808 (2.319–3.400)	3.9192×10^{-26}
GRS	1.777 (1.415–2.231)	7.4856×10^{-7}
AUROC	0.705 (0.678–0.733)	2.6415×10^{-40}

ORs with 95% CIs and *p* values were derived from logistic regression. CI, confidence interval; AUROC, area under the ROC curve.

Range of BMI (kg/m^2): low, <24.197; high, ≥24.197

Range of OSS: low, <1.322; high, ≥1.322

Range of GRS: low, <1.556; high, ≥1.556
